# 5-Phenyl-7,8-dihydro-1,3-dioxano[4,5-*g*]isoquinoline

**DOI:** 10.1107/S1600536808035009

**Published:** 2008-11-08

**Authors:** Jiu-Ming Li, Dong Liang

**Affiliations:** a College of Chemistry, Inner Mongolia University for Nationalities, Tongliao 028043, People’s Republic of China; b Qingdao DIC Finechemicals Co. Ltd, Qingdao 266101, People’s Republic of China

## Abstract

In the title compound, C_16_H_13_NO_2_, the two benzene rings make a dihedral angle of 55.5 (2)°. The crystal packing is stabilized by inter­molecular C—H⋯O hydrogen bonds and weak π–π stacking inter­actions [centroid–centroid distance = 3.595 (3)Å], linking the mol­ecules into ladders of inversion dimers.

## Related literature

For details of the biological activities of isoquinolinone compounds, see: Bentley (2000 ▶); Jayaraman *et al.* (2002 ▶). For the Bischler–Napieralski reaction, see: Bischler & Napieralski (1893 ▶). For bond-length data, see: Allen *et al.* (1987 ▶).
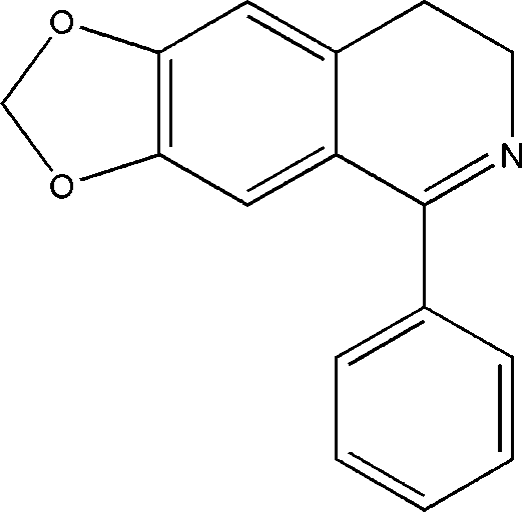


## Experimental

### 

#### Crystal data

C_16_H_13_NO_2_
*M* *_r_* = 251.27
Triclinic, 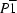

*a* = 8.5005 (17) Å
*b* = 8.5297 (17) Å
*c* = 10.143 (2) Å
α = 109.07 (3)°
β = 109.44 (2)°
γ = 99.70 (3)°
*V* = 622.9 (2) Å^3^
*Z* = 2
Mo *K*α radiation
μ = 0.09 mm^−1^
*T* = 293 (2) K
0.28 × 0.10 × 0.08 mm

#### Data collection

Rigaku R-AXIS RAPID IP area-detector diffractometer
Absorption correction: multi-scan (*ABSCOR*; Higashi, 1995 ▶) *T*_min_ = 0.976, *T*_max_ = 0.993
4801 measured reflections
2145 independent reflections
1275 reflections with *I* > 2σ(*I*)
*R*_int_ = 0.037

#### Refinement

*R*[*F* ^2^ > 2σ(*F* ^2^)] = 0.041
*wR*(*F* ^2^) = 0.143
*S* = 1.13
2145 reflections
173 parameters
H-atom parameters constrained
Δρ_max_ = 0.20 e Å^−3^
Δρ_min_ = −0.20 e Å^−3^

### 

Data collection: *RAPID-AUTO* (Rigaku, 2004 ▶); cell refinement: *RAPID-AUTO*; data reduction: *RAPID-AUTO*; program(s) used to solve structure: *SHELXTL* (Sheldrick, 2008 ▶); program(s) used to refine structure: *SHELXTL*; molecular graphics: *SHELXTL*; software used to prepare material for publication: *SHELXTL*.

## Supplementary Material

Crystal structure: contains datablocks I, global. DOI: 10.1107/S1600536808035009/hg2432sup1.cif

Structure factors: contains datablocks I. DOI: 10.1107/S1600536808035009/hg2432Isup2.hkl

Additional supplementary materials:  crystallographic information; 3D view; checkCIF report

## Figures and Tables

**Table 1:** Hydrogen-bond geometry (Å, °)

*D*—H⋯*A*	*D*—H	H⋯*A*	*D*⋯*A*	*D*—H⋯*A*
C3—H3*A*⋯O2^i^	0.93	2.55	3.465 (4)	169

Symmetry code: (i) 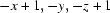.

**Fig. 1.**
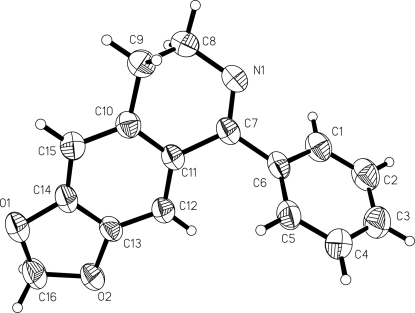
The molecular structure of (I), with atom labels and 40% probability displacement ellipsoids for non-H atoms.

## supplementary crystallographic information

### Comment

Isoquinolinones are important compounds from both the synthetic and applied points of view. Their structures are incorporated in several alkaloids (Bentley, 20000 and other pharmacologically important compounds (Jayaraman *et al.*, 2002). Of the variety of methods that have been developed for the synthesis of the isoquinoline ring system, the most commonly used procedure is the Bischler-Napieralski reaction (Bischler & Napieralski, 1893). We now wish to report an effective Bischler-Napieralski procedure for the synthesis of 1,2,3,4-tetrahydro-6,7-dimethoxy-1-phenylisoquinoline the title compound (I) and report its crystal structure here.

In compound (I), all bond lengths in the molecular are normal (Allen *et al.*, 1987). The benzene ring C10–C15 and bonded atoms C7, C9, O1 and O2 are coplanar, the largest deviation from the mean plane being 0.039 (2)Å for atom O1. The other benzene ring, C1–C6, and bonded atoms C7 are also coplanar, the largest deviation from the mean plane being 0.032 (2)Å. The two benzene rings make a dihedral angle of 55.5 (2)°.

The relatively short distance of 3.595 (3) between the centroids of benzene ring C10—C15 and 1,3-dioxole ring C13/C14/C16/O1/O2 [at -*x*,1 - *y*,-*z*] indicates the presence of weak π-π interactions, The crystal packing is stabilized by intermolecular C—H···O hydrogen bonds, linking the molecules into ladders of dimers.

### Experimental

The title compound was synthesized by following Bischler-Napieralski procedures: 0.01 mol N-[2-(3, 4-methylenedioxy)phenyl]benzamide (synthesized by β-(3, 4-methylenedioxy)phenethylamine, Benzoyl chloride and Et_3_N) was dissolved in 20 ml CH_3_CN, 5 g POCl_3_ was added dropwise, the mixture was refluxed under N_2_ for 5 h, after cooled the volatiles were evaporated under vacuum, then water was added and adjusted the pH to 8, after extracted with CH_2_Cl_2_, the organic layers was washed with saturated NaCl and dried with Na_2_SO_4_, the product was isolated by evaporation of the solvent and recrystalization, 2.31 g, Yield: 92%. Single crystals suitable for X-ray measurements were obtained by recrystallization from ethyl acetate at room temperature.

### Refinement

H atoms were positioned geometrically and refined using a riding model, with C—H = 0.96 Å, with *U*_iso_(H) = 1.2 times *U*_eq_(C).

### Crystal data

**Table:**

C_16_H_13_NO_2_	*Z* = 2
*M**_r_* = 251.27	*F*_000_ = 264
Triclinic, *P*1	*D*_x_ = 1.340 Mg m^−^^3^
Hall symbol: -P 1	Mo *K*α radiation λ = 0.71073 Å
*a* = 8.5005 (17) Å	Cell parameters from 1451 reflections
*b* = 8.5297 (17) Å	θ = 2.5–23.4º
*c* = 10.143 (2) Å	µ = 0.09 mm^−^^1^
α = 109.07 (3)º	*T* = 293 (2) K
β = 109.44 (2)º	Block, colorless
γ = 99.70 (3)º	0.28 × 0.10 × 0.08 mm
*V* = 622.9 (2) Å^3^	

### Data collection

**Table:**

Rigaku R-AXIS RAPID IP area-detector diffractometer	2145 independent reflections
Radiation source: Rotating Anode	1275 reflections with *I* > 2σ(*I*)
Monochromator: graphite	*R*_int_ = 0.037
*T* = 293(2) K	θ_max_ = 25.0º
ω oscillation scans	θ_min_ = 3.1º
Absorption correction: multi-scan(ABSCOR; Higashi, 1995)	*h* = −10→10
*T*_min_ = 0.976, *T*_max_ = 0.993	*k* = −10→10
4801 measured reflections	*l* = −11→12

### Refinement

**Table:**

Refinement on *F*^2^	Hydrogen site location: inferred from neighbouring sites
Least-squares matrix: full	H-atom parameters constrained
*R*[*F*^2^ > 2σ(*F*^2^)] = 0.041	*w* = 1/[σ^2^(*F*_o_^2^) + (0.0514*P*)^2^ + 0.1397*P*] where *P* = (*F*_o_^2^ + 2*F*_c_^2^)/3
*wR*(*F*^2^) = 0.144	(Δ/σ)_max_ < 0.001
*S* = 1.13	Δρ_max_ = 0.20 e Å^−^^3^
2145 reflections	Δρ_min_ = −0.20 e Å^−^^3^
173 parameters	Extinction correction: SHELXTL (Sheldrick, 2008), Fc^*^=kFc[1+0.001xFc^2^λ^3^/sin(2θ)]^-1/4^
Primary atom site location: structure-invariant direct methods	Extinction coefficient: 0.048 (8)
Secondary atom site location: difference Fourier map	

### Special details

**Table:**

Geometry. All e.s.d.'s (except the e.s.d. in the dihedral angle between two l.s. planes) are estimated using the full covariance matrix. The cell e.s.d.'s are taken into account individually in the estimation of e.s.d.'s in distances, angles and torsion angles; correlations between e.s.d.'s in cell parameters are only used when they are defined by crystal symmetry. An approximate (isotropic) treatment of cell e.s.d.'s is used for estimating e.s.d.'s involving l.s. planes.
Refinement. Refinement of *F*^2^ against ALL reflections. The weighted *R*-factor *wR* and goodness of fit *S* are based on *F*^2^, conventional *R*-factors *R* are based on *F*, with *F* set to zero for negative *F*^2^. The threshold expression of *F*^2^ > σ(*F*^2^) is used only for calculating *R*-factors(gt) *etc*. and is not relevant to the choice of reflections for refinement. *R*-factors based on *F*^2^ are statistically about twice as large as those based on *F*, and *R*- factors based on ALL data will be even larger.

### Fractional atomic coordinates and isotropic or equivalent isotropic displacement parameters (Å^2^)

**Table:**

	*x*	*y*	*z*	*U*_iso_*/*U*_eq_	
O1	0.9151 (2)	0.7157 (2)	1.18121 (17)	0.0688 (6)	
O2	0.6920 (2)	0.4574 (2)	1.01260 (17)	0.0656 (6)	
N1	0.8054 (3)	0.6182 (3)	0.5069 (2)	0.0638 (6)	
C1	0.5553 (3)	0.2889 (4)	0.3031 (3)	0.0614 (7)	
H1B	0.5296	0.3808	0.2795	0.074*	
C2	0.4741 (4)	0.1204 (4)	0.1932 (3)	0.0734 (8)	
H2A	0.3938	0.0996	0.0961	0.088*	
C3	0.5097 (4)	−0.0172 (4)	0.2247 (3)	0.0772 (9)	
H3A	0.4550	−0.1308	0.1496	0.093*	
C4	0.6281 (4)	0.0149 (4)	0.3695 (3)	0.0692 (8)	
H4A	0.6521	−0.0779	0.3924	0.083*	
C5	0.7107 (3)	0.1825 (3)	0.4798 (3)	0.0579 (7)	
H5A	0.7914	0.2022	0.5765	0.069*	
C6	0.6756 (3)	0.3230 (3)	0.4491 (2)	0.0511 (6)	
C7	0.7705 (3)	0.5073 (3)	0.5614 (2)	0.0521 (6)	
C8	0.9013 (4)	0.7997 (3)	0.6168 (3)	0.0679 (8)	
H8A	0.8187	0.8570	0.6406	0.081*	
H8B	0.9573	0.8600	0.5702	0.081*	
C9	1.0385 (4)	0.8139 (3)	0.7632 (3)	0.0604 (7)	
H9A	1.1279	0.7664	0.7424	0.072*	
H9B	1.0943	0.9354	0.8351	0.072*	
C10	0.9508 (3)	0.7137 (3)	0.8300 (2)	0.0497 (6)	
C11	0.8169 (3)	0.5585 (3)	0.7272 (2)	0.0468 (6)	
C12	0.7220 (3)	0.4618 (3)	0.7802 (2)	0.0490 (6)	
H12A	0.6326	0.3575	0.7130	0.059*	
C13	0.7659 (3)	0.5266 (3)	0.9336 (2)	0.0492 (6)	
C14	0.8994 (3)	0.6795 (3)	1.0347 (2)	0.0510 (6)	
C15	0.9955 (3)	0.7752 (3)	0.9871 (2)	0.0525 (6)	
H15A	1.0871	0.8771	1.0566	0.063*	
C16	0.7728 (4)	0.5854 (4)	1.1664 (3)	0.0713 (8)	
H16A	0.8159	0.5318	1.2363	0.086*	
H16B	0.6882	0.6379	1.1918	0.086*	

### Atomic displacement parameters (Å^2^)

**Table:**

	*U*^11^	*U*^22^	*U*^33^	*U*^12^	*U*^13^	*U*^23^
O1	0.0833 (14)	0.0751 (12)	0.0399 (9)	0.0155 (10)	0.0240 (9)	0.0202 (8)
O2	0.0861 (14)	0.0677 (11)	0.0470 (10)	0.0158 (10)	0.0377 (9)	0.0221 (9)
N1	0.0805 (16)	0.0671 (14)	0.0497 (12)	0.0199 (12)	0.0329 (11)	0.0267 (11)
C1	0.0513 (15)	0.0779 (18)	0.0514 (15)	0.0196 (13)	0.0185 (12)	0.0255 (14)
C2	0.0562 (17)	0.091 (2)	0.0482 (15)	0.0073 (15)	0.0087 (12)	0.0213 (15)
C3	0.080 (2)	0.0689 (18)	0.0552 (16)	0.0037 (16)	0.0187 (14)	0.0116 (14)
C4	0.079 (2)	0.0613 (16)	0.0564 (16)	0.0162 (14)	0.0246 (14)	0.0179 (13)
C5	0.0633 (16)	0.0625 (15)	0.0404 (13)	0.0169 (13)	0.0184 (11)	0.0168 (12)
C6	0.0522 (15)	0.0630 (15)	0.0383 (12)	0.0164 (12)	0.0232 (10)	0.0173 (11)
C7	0.0588 (16)	0.0615 (15)	0.0433 (12)	0.0229 (12)	0.0272 (11)	0.0221 (12)
C8	0.087 (2)	0.0622 (16)	0.0626 (16)	0.0183 (14)	0.0396 (15)	0.0296 (14)
C9	0.0646 (17)	0.0594 (15)	0.0592 (15)	0.0142 (12)	0.0327 (13)	0.0223 (12)
C10	0.0498 (14)	0.0533 (13)	0.0471 (13)	0.0170 (11)	0.0226 (11)	0.0190 (11)
C11	0.0517 (14)	0.0525 (13)	0.0393 (12)	0.0180 (11)	0.0220 (10)	0.0186 (10)
C12	0.0533 (15)	0.0522 (13)	0.0427 (12)	0.0180 (11)	0.0221 (10)	0.0178 (11)
C13	0.0587 (15)	0.0553 (14)	0.0405 (12)	0.0199 (11)	0.0273 (11)	0.0202 (11)
C14	0.0596 (16)	0.0587 (14)	0.0359 (12)	0.0255 (12)	0.0197 (11)	0.0178 (11)
C15	0.0507 (14)	0.0560 (14)	0.0458 (13)	0.0150 (11)	0.0187 (11)	0.0171 (11)
C16	0.087 (2)	0.0816 (19)	0.0449 (14)	0.0216 (16)	0.0341 (14)	0.0215 (14)

### Geometric parameters (Å, °)

**Table:**

O1—C14	1.370 (3)	C7—C11	1.481 (3)
O1—C16	1.427 (3)	C8—C9	1.504 (4)
O2—C13	1.381 (3)	C8—H8A	0.9700
O2—C16	1.421 (3)	C8—H8B	0.9700
N1—C7	1.283 (3)	C9—C10	1.499 (3)
N1—C8	1.465 (3)	C9—H9A	0.9700
C1—C2	1.375 (4)	C9—H9B	0.9700
C1—C6	1.391 (3)	C10—C11	1.391 (3)
C1—H1B	0.9300	C10—C15	1.394 (3)
C2—C3	1.368 (4)	C11—C12	1.406 (3)
C2—H2A	0.9300	C12—C13	1.359 (3)
C3—C4	1.380 (4)	C12—H12A	0.9300
C3—H3A	0.9300	C13—C14	1.376 (3)
C4—C5	1.372 (3)	C14—C15	1.364 (3)
C4—H4A	0.9300	C15—H15A	0.9300
C5—C6	1.387 (3)	C16—H16A	0.9700
C5—H5A	0.9300	C16—H16B	0.9700
C6—C7	1.487 (3)		
			
C14—O1—C16	105.44 (18)	C10—C9—C8	108.3 (2)
C13—O2—C16	105.24 (18)	C10—C9—H9A	110.0
C7—N1—C8	117.11 (19)	C8—C9—H9A	110.0
C2—C1—C6	120.6 (3)	C10—C9—H9B	110.0
C2—C1—H1B	119.7	C8—C9—H9B	110.0
C6—C1—H1B	119.7	H9A—C9—H9B	108.4
C3—C2—C1	120.9 (3)	C11—C10—C15	121.1 (2)
C3—C2—H2A	119.6	C11—C10—C9	116.9 (2)
C1—C2—H2A	119.6	C15—C10—C9	122.0 (2)
C2—C3—C4	119.1 (3)	C10—C11—C12	120.3 (2)
C2—C3—H3A	120.5	C10—C11—C7	117.7 (2)
C4—C3—H3A	120.5	C12—C11—C7	121.8 (2)
C5—C4—C3	120.5 (3)	C13—C12—C11	117.4 (2)
C5—C4—H4A	119.7	C13—C12—H12A	121.3
C3—C4—H4A	119.7	C11—C12—H12A	121.3
C4—C5—C6	120.9 (2)	C12—C13—C14	121.9 (2)
C4—C5—H5A	119.6	C12—C13—O2	128.3 (2)
C6—C5—H5A	119.6	C14—C13—O2	109.77 (19)
C5—C6—C1	118.0 (2)	C15—C14—O1	127.9 (2)
C5—C6—C7	122.7 (2)	C15—C14—C13	122.1 (2)
C1—C6—C7	119.0 (2)	O1—C14—C13	110.0 (2)
N1—C7—C11	122.4 (2)	C14—C15—C10	117.2 (2)
N1—C7—C6	116.7 (2)	C14—C15—H15A	121.4
C11—C7—C6	120.8 (2)	C10—C15—H15A	121.4
N1—C8—C9	112.5 (2)	O2—C16—O1	108.54 (19)
N1—C8—H8A	109.1	O2—C16—H16A	110.0
C9—C8—H8A	109.1	O1—C16—H16A	110.0
N1—C8—H8B	109.1	O2—C16—H16B	110.0
C9—C8—H8B	109.1	O1—C16—H16B	110.0
H8A—C8—H8B	107.8	H16A—C16—H16B	108.4
			
C6—C1—C2—C3	−0.1 (4)	N1—C7—C11—C10	−22.2 (4)
C1—C2—C3—C4	0.5 (5)	C6—C7—C11—C10	159.5 (2)
C2—C3—C4—C5	−0.8 (5)	N1—C7—C11—C12	153.3 (3)
C3—C4—C5—C6	0.9 (4)	C6—C7—C11—C12	−25.0 (4)
C4—C5—C6—C1	−0.5 (4)	C10—C11—C12—C13	0.3 (4)
C4—C5—C6—C7	−175.7 (2)	C7—C11—C12—C13	−175.0 (2)
C2—C1—C6—C5	0.1 (4)	C11—C12—C13—C14	−0.9 (4)
C2—C1—C6—C7	175.6 (2)	C11—C12—C13—O2	179.4 (2)
C8—N1—C7—C11	2.5 (4)	C16—O2—C13—C12	−173.7 (3)
C8—N1—C7—C6	−179.1 (2)	C16—O2—C13—C14	6.5 (3)
C5—C6—C7—N1	142.5 (3)	C16—O1—C14—C15	174.1 (3)
C1—C6—C7—N1	−32.7 (3)	C16—O1—C14—C13	−5.3 (3)
C5—C6—C7—C11	−39.2 (3)	C12—C13—C14—C15	0.0 (4)
C1—C6—C7—C11	145.6 (2)	O2—C13—C14—C15	179.8 (2)
C7—N1—C8—C9	37.6 (3)	C12—C13—C14—O1	179.4 (2)
N1—C8—C9—C10	−56.4 (3)	O2—C13—C14—O1	−0.8 (3)
C8—C9—C10—C11	37.8 (3)	O1—C14—C15—C10	−178.0 (2)
C8—C9—C10—C15	−139.8 (3)	C13—C14—C15—C10	1.3 (4)
C15—C10—C11—C12	1.0 (4)	C11—C10—C15—C14	−1.8 (4)
C9—C10—C11—C12	−176.6 (2)	C9—C10—C15—C14	175.7 (2)
C15—C10—C11—C7	176.5 (2)	C13—O2—C16—O1	−9.7 (3)
C9—C10—C11—C7	−1.1 (3)	C14—O1—C16—O2	9.3 (3)

### Hydrogen-bond geometry (Å, °)

**Table:**

*D*—H···*A*	*D*—H	H···*A*	*D*···*A*	*D*—H···*A*
C3—H3A···O2^i^	0.93	2.55	3.465 (4)	169

Symmetry codes: (i) −*x*+1, −*y*, −*z*+1.

## References

[bb1] Allen, F. H., Kennard, O., Watson, D. G., Brammer, L., Orpen, A. G. & Taylor, R. (1987). *J. Chem. Soc. Perkin Trans. 2*, pp. S1–19.

[bb2] Bentley, K. B. (2000). *Nat. Prod. Rep.* **17**, 247–268. 10.1039/a900251k 10888012

[bb3] Bischler, A. & Napieralski, B. (1893). *Chem. Ber.* **26**, 1903.

[bb4] Higashi, T. (1995). *ABSCOR* Rigaku Corporation, Tokyo, Japan.

[bb5] Jayaraman, M., Fox, B. M., Hollingshead, M., Kohlhagen, G., Pommier, Y. & Cushman, M. (2002). *J. Med. Chem.* **44**, 242–249. 10.1021/jm000498f 11754595

[bb6] Rigaku (2004). *RAPID-AUTO* Rigaku Corporation, Takyo, Japan.

[bb7] Sheldrick, G. M. (2008). *Acta Cryst.* A**64**, 112–122. 10.1107/S0108767307043930 18156677

